# Proteomics Insights Into the Molecular Basis of SARS-CoV-2 Infection: What We Can Learn From the Human Olfactory Axis

**DOI:** 10.3389/fmicb.2020.02101

**Published:** 2020-09-22

**Authors:** Mercedes Lachén-Montes, Fernando J. Corrales, Joaquín Fernández-Irigoyen, Enrique Santamaría

**Affiliations:** ^1^Clinical Neuroproteomics Unit, Proteomics Platform, Navarrabiomed, Complejo Hospitalario de Navarra (CHN), Universidad Pública de Navarra (UPNA), Pamplona, Spain; ^2^IdiSNA, Navarra Institute for Health Research, Pamplona, Spain; ^3^Proteored-Instituto de Salud Carlos III (ISCIII), Madrid, Spain; ^4^Proteomics Unit, National Centre for Biotechnology, Madrid, Spain

**Keywords:** proteomics, coronavirus infectious disease 2019, severe acute respiratory syndrome coronavirus-2, smell, mass-spectrometry

## Abstract

Like other RNA viruses, severe acute respiratory syndrome coronavirus 2 (SARS-CoV-2) replicates in host cells, continuously modulating the molecular environment. It encodes 28 multifunctional proteins that induce an imbalance in the metabolic and proteostatic homeostasis in infected cells. Recently, proteomic approaches have allowed the evaluation of the impact of SARS-CoV-2 infection in human cells. Here, we discuss the current use of proteomics in three major application areas: (i) virus-protein interactomics, (ii) differential proteotyping to map the virus-induced changes in different cell types, and (iii) diagnostic methods for coronavirus infectious disease 2019 (COVID-19). Since the nasal cavity is one of the entry sites for SARS-CoV-2, we will also discuss the potential application of olfactory proteomics to provide novel insights into the olfactory dysfunction triggered by SARS-CoV-2 in patients with COVID-19.

## Introduction

Since the outbreak of the pandemic in Wuhan (China) in December 2019, coronavirus infectious disease 2019 (COVID-19) has spread globally, causing a crisis not only in our healthcare systems but also in the world economy (Dong et al., 2020; Wu et al., 2020c; Zhou et al., 2020). Among other symptoms, patients with COVID-19 present with anosmia, fever, cough, and dyspnea (Parma et al., 2020; Wu et al., 2020b). Unfortunately, specific treatments are still lacking and the scientific community is struggling to develop new therapeutic combinations and discover effective vaccines.

The recently emerged severe acute respiratory syndrome coronavirus 2 (SARS-CoV-2) belongs to the family of betacoronaviruses and contains a single-stranded RNA of approximately 30 kb in length (Wu et al., 2020a). Altogether, the subgroups of coronaviruses were thought to exclusively infect animals (Ng and Hiscox, 2020); however, severe acute respiratory syndrome coronavirus (SARS-CoV) and Middle East respiratory syndrome-related coronavirus caused severe respiratory illness in humans in 2002 and 2012, respectively (Peiris et al., 2004; Kupferschmidt, 2014). In particular, SARS-CoV-2 has infected more than 5 million individuals worldwide, causing more than 300,000 deaths at the time of writing.

The process of SARS-CoV-2 infection begins with the binding of the viral glycoprotein spike S protein to angiotensin-converting enzyme 2 (ACE2), whose receptor is expressed on the human airway epithelium. After fusion occurs, the type II transmembrane serine protease (TMPRSS2) present on the cell surface clears ACE2 and activates the receptor-attached spike-like S protein (Hoffmann et al., 2020), leading to conformational changes necessary for virus entry. After viral RNA release in the cytoplasm, the genomic RNA is translated into the viral replicase polyproteins pp1 and 1ab. Then, the polymerase produces a series of subgenomic mRNAs, which are translated into new structural proteins and translocated from the endoplasmic reticulum to the Golgi apparatus, where the new virions are assembled. At this point, there remain significant gaps in our knowledge concerning the host effectors involved in viral replication and infection. Following examination of the SARS-CoV-2 transcriptome (Ferretti et al., 2020; Kim et al., 2020), the use of proteomic strategies has emerged as a valuable tool to cope with this viral sprout. In fact, proteomic methods allowed the characterization and understanding of SARS-CoV during the severe acute respiratory syndrome (SARS) outbreak in 2003, enabling the description of virus protein sequences and antigenic virus proteins and the identification of ACE2 as the human receptor for SARS-CoV (Krokhin et al., 2003; Li et al., 2003; Ying et al., 2004). Consequently, the application of proteomic workflows may be highly beneficial to increase our understanding of the pathogenesis of COVID-19, to identify potential therapeutic targets, and even to develop fast and effective diagnostic tests (Struwe et al., 2020; Whetton et al., 2020). In this mini-review, we focus on different proteomic methodologies currently used to decipher novel SARS-CoV-2 targets and potential COVID-19 biomarkers (Table 1). Given that the nasal cavity is one of the entry sites for SARS-CoV-2, we will also discuss the deployment of olfactory proteomics to unravel cell-signaling networks that are disrupted in olfactory peripheral areas that may potentially explain the early loss of smell observed in patients with COVID-19.

**Table 1:** Compilation of recent research using proteomic approaches in a SARS-CoV-2 context.

Objective	Authors	Sample	Technological platform	Findings
SARS-CoV-2 interactome	Liang et al. (2020)	SARS-CoV-2 proteins and HEK293 cells	LC-MS/MS (Q-Exactive Orbitrap)	Identification of 332 high-confidence SARS-CoV-2 human PPIs; 62 of them have drugs/preclinical molecules that modulate them
	Gordon et al. (2020)	SARS-CoV-2 proteins and HEK293 cells	LC-MS/MS (Q-Exactive Plus)	Identification of 631 SARS-CoV-2 human PPIs (deregulation in translation, degradation, inflammatory, and innate immune system)
	Stukalov et al. (2020)	SARS-CoV-2 proteins and A549 cells	LC-MS/MS (Q-Exactive HF-X)	Identification of 1,484 interactions between 1,086 cellular proteins and 24 SARS-CoV-2 targets involving innate immunity and stress response components, and DNA damage response mediators
SARS-CoV-2 *in vitro* models	Bojkova et al. (2020)	Caco-212 cells (colon)	LC-MS/MS (Q-Exactive HF)	Proteins
	Grenga et al. (2020)	Vero cells (renal)	LC-MS/MS (Q-Exactive HF)	Alteration in membrane trafficking, pre-processing in ER and regulation of mRNA processing/splicing *via* spliceosome
	Davidson et al. (2020)	Vero cells (renal)	RNAseq + LC-MS/MS (Q-Exactive HF)	Identification of an 8 amino acid deletion in the S glycoprotein that potentially affects protein cleavage, cell tropism, and infectivity
	Appelberg et al. (2020)	HuH7 cells (hepatic)	RNAseq + LC-MS/MS (Orbitrap Fusion Lumos)	Proteo-transcriptomic analysis has identified disruptions in ErbB, HIF-1, mTOR, and TNF signaling
Covid-19 pathology study and diagnosis	Ihling et al. (2020)	Gargle	LC-MS/MS (Orbitrap-HCD)	An MS method that specifically detects unique peptides from SARS-CoV-2 nucleoprotein from gargle samples
	Bezstarosti et al. (2020)	Vero cells (renal)	LC-MS/MS (Orbitrap Eclipse Tribrid)	A parallel reaction monitoring approach to detect the SARS-CoV-2 nucleocapsid protein in the mid-attomole range
	Nikolaev et al. (2020)	Nasopharynx	LC-MS/MS (TIMS-TOF)	Confident identification of the SARS-CoV-2 N protein in patients with low viral load
	Wang et al. (2020)	Serum	Microarray (n.d.)	Antibodies commercially available for SARS-CoV-1 proteins can also target SARS-CoV-2 proteins
	Zhang et al. (2020)	Serum	Microarray (GenePix 4300A microarray)	The S protein S1-RBD might be the optimal antigen for IgM antibody detection, while the S protein extracellular domain would be the optimal antigen for both IgM and IgG antibody detection
	Shen et al. (2020)	Serum	LC-MS/MS (Q-Exactive HF-X)	Deregulation of 93 proteins and 204 metabolites revealed metabolic and immune dysregulation in patients with COVID-19
	Liang et al. (2020)	PBMCs	LC-MS/MS (Q-Exactive Orbitrap)	Two hundred and twenty differential expressed proteins involved in neutrophil chemotaxis, type I interferon signaling pathway, inflammatory response, and antigen processing and presentation in PBMCs from patients with COVID-19
	Messner et al. (2020)	Serum	LC-MS/MS (TripleTOF 6600)	Twenty-seven potential serum biomarkers that distinguish mild and severe forms of COVID-19
	Akgun et al. (2020)	Nasopharyngeal swab	LC-MS/MS [Xevo G2-XS QTOF (SONAR)]	Seventeen differentially expressed proteins across COVID-19 samples involved in neutrophil degranulation and innate immune responses
	Liang et al. (2020)	Urine	LC-MS/MS (Orbitrap Fusion Lumos)	A severe proteomic imbalance among COVID-19 samples with an impact on immune response, complement activation, platelet degranulation, lipoprotein metabolic process, and response to hypoxia

ER, endoplasmic reticulum; ErbB, epidermal growth factor receptor; HIF-1, hypoxia-inducible factor 1; IgG, immunoglobulin G; IgM, immunoglobulin M; LC-MS/MS, liquid chromatography with tandem mass spectrometry; mTOR, mammalian target of rapamycin; n.d., not determined; PBMC, peripheral blood mononuclear cell; PPI, protein-protein interaction; S1-RBD, subunit 1 receptor binding domain; TIMS-TOF, trapped ion mobility spectrometry-time of flight; TNF, tumor necrosis factor.

## Elucidation of the SARS-CoV-2 Interactome

It is well known that viral proteomes constitute different combinations of protein-protein interactions (PPIs) to accomplish a great variety of vital functions during the viral cycle. However, the study of protein interactions is challenging because of their special physical properties and transitory character. To study protein complexes, a combination of cross-linking and liquid chromatography-tandem mass spectrometry (LC-MS/MS) approaches is commonly used (Haupt et al., 2017), and these techniques have been used recently for the analysis of SARS-CoV-2 PPIs. The 30 kb SARS-CoV-2 genome encodes for 14 major open reading frames (ORFs) that are further processed into 28 proteins: (i) glycoprotein spike (S), membrane (M), envelope (E), and nucleocapsid (N) structural proteins, (ii) accessory proteins (ORF10, ORF9a, ORF9b, ORF8, ORF7a, ORF7b, ORF6, and ORF3a), and (iii) 16 non-structural proteins (nsp1–nsp16; Wu et al., 2020a). Liang et al. (2020) characterized intraviral PPIs using genome-wide yeast-two hybrid and co-immunoprecipitation, revealing 58 viral PPIs. Interestingly, some viral proteins showed self-association forms, including proteins M, N, and E, some non-structural proteins (nsp2, nsp5, and nsp8), and some accessory proteins (ORF6, ORF7a, ORF7b, ORF9b, and ORF10), suggesting their important roles in viral replication and immune evasion. In this context, it is important to note that various disadvantages have been addressed in the study of PPIs, such as the lack of essential post-translational modifications for protein interactions that do not occur in yeast or the potential toxicity of some proteins in this system (Van Criekinge and Beyaert, 1999).

Since no specific therapeutics are currently approved for SARS-CoV-2, it is essential to increase knowledge regarding the interaction landscape between viral and human proteins. Taking into account the drawbacks concerning the overexpression of exogenous proteins in cell systems such as protein misfolding, mislocalization, or misregulations (Dunham et al., 2012), the combination of affinity purification and mass spectrometry (AP-LC/MS) represents a feasible approach to obtain high-quality maps of human protein interactomes in a reproducible way (Varjosalo et al., 2013). Gordon et al. (2020) have identified 332 protein interactions between SARS-CoV-2 and human proteins. The SARS-CoV-2 human interactome has been shown to impact functions such as DNA replication, epigenetic and gene expression regulation, vesicle trafficking, lipid metabolism, and RNA processing, among others (Gordon et al., 2020). Importantly, SARS-CoV-2 viral proteins target proteins involved in the innate immune system. For instance, Nsp13 interacts with multiple proteins from the transducin-like enhancer of split (TLE) family, which modulates the nuclear factor (NF)-κB response. Accordingly, interactions between human inflammatory and innate immune key players, such as NF-κB repressing factor [NKRF; a repressor of interleukin (IL)-8 and IL-6 production], C1QBP (involved in complement C1 activation), and a great variety of viral non-structural proteins (nsp9, nsp10, etc.), have also been described by Liang et al. (2020) using AP-LC/MS analysis, explaining the high inflammatory response of patients with COVID-19 (Tay et al., 2020). In this case, MS analysis identified 251 human proteins as SARS-CoV-2 interactors. Among others, ribosomal proteins and proteasome-related proteins were identified as viral interactors, indicating alterations in the host gene translation, protein folding, and the degradation pathways. In both studies, the authors overexpressed the SARS-CoV-2 genes with a flag epitope into HEK293 cells. However, the characterized interactome partially differs, probably because of the experimental settings applied (i.e., the timing of cell lysis). Finally, the multilevel proteomic approach described by Stukalov et al. (2020), through the SARS-CoV and SARS-CoV-2 interactome in a lung-derived human cell line (A549), has revealed specific characteristics in the pathogenicity and transmission capabilities of each strain. Moreover, this interactome profiling has allowed the identification of potential targets for SARS-CoV-2 antiviral therapies (Stukalov et al., 2020).

## Proteomic Profiling of SARS-CoV-2-Infected Host Cells

High-throughput techniques have also been applied to increase biological knowledge of the molecular imbalance triggered by SARS-CoV-2 infection using *in vitro* models. Analyzing the quantitative translatome and proteome profiles of the Caco-2 cellular response on infection with SARS-CoV-2 (Bojkova et al., 2020), only minor changes were observed at early infection time points. However, alterations in spliceosome components and carbon metabolism were observed at 24 h post infection (h.p.i.). In this context, the authors proposed the use of specific inhibitors of both pathways as potential therapeutic agents for SARS-CoV-2 infection. Consistent with these data, alterations in the same biological routes together with deregulation in protein pre-processing, vacuole formation, and viral budding routes have been detected in SARS-CoV-2-infected Vero cells (Grenga et al., 2020). Moreover, multi-omic characterization at the level of the complete genome, proteome, and phosphoproteome was performed using the same cell line, revealing new and critical aspects of SARS-CoV-2 glycoprotein S (Davidson et al., 2020). Finally, Appelberg et al. (2020) combined two high-throughput techniques analyzing the transcriptome and proteome of Huh7 cells upon SARS-CoV-2 infection at 24, 48, and 72 h.p.i. Together with alterations in the thrombotic and metabolic pathways (complement cascades and glycolysis), the mammalian target of rapamycin (mTOR)/hypoxia-inducible factor 1 (HIF-1) signaling route was proposed as a potential therapeutic target against SARS-CoV-2 infection (Appelberg et al., 2020). All differential molecules characterized in SARS-CoV-2-infected cells have extended our knowledge about the metabolic imbalance induced during the viral infection. However, the molecular repertoires differ significantly across studies. This variability is likely associated with the experimental designs applied (different origin of the cell lines, multiplicity of infection, and time course used) and the heterogeneity of the analytical workflows used (protein precipitation, digestion and fractionation protocols, and MS workflows; Table 1). Although differential proteotyping has contributed to a better understanding about SARS-CoV-2 infection, more efforts are needed to decipher whether the differentially expressed proteins may be considered SARS-CoV-2 targets involved in the modulation of the host metabolism or are part of the molecular events triggered by the cell to interfere with viral propagation.

## Clinical Proteomics in Covid-19

Although the genome/proteome characterization of SARS-CoV-2 provides valuable information regarding the virus structure and protein interactions, understanding how the infection alters the metabolic homeostasis in patients with COVID-19 may provide resources for novel diagnostic and therapeutic interventions. The diagnostic potential of proteomics has already been demonstrated by a shotgun proteomic workflow, in which SARS-CoV-2 unique peptides were identified in gargle samples from patients with COVID-19 (Ihling et al., 2020) and SARS-CoV-2 peptides have been identified in the mid-attomole range by parallel reaction monitoring (Bezstarosti et al., 2020). Moreover, a rapid MS protocol has been recently developed to detect peptides corresponding to the viral nucleocapsid N protein from nasopharyngeal epithelial swab samples in less than 1 h (Nikolaev et al., 2020). In order to improve serology tests for rapid COVID-19 screening, antibody microarrays detecting SARS-CoV-2 antigens have also been developed, mapping the humoral response of patients with COVID-19 (Wang et al., 2020; Zhang et al., 2020). Also, Shen et al. (2020) have combined isobaric proteomics and untargeted metabolomics with the aim of analyzing molecular perturbances occurring in the sera of patients with SARS-CoV-2. This study has shown a dysregulation in lipid metabolism due to changes in multiple apolipoproteins and steroid hormones, and activation of acute-phase proteins and the complement system. On the other hand, Liang et al. (2020) performed a labeling quantitative LC-MS/MS analysis in peripheral blood mononuclear cells from patients with COVID-19, revealing an imbalance in neutrophil activation, type I interferon signaling, inflammation, and antigen processing (Liang et al., 2020). Accordingly, recent proteomic studies have also demonstrated alterations in neutrophil and platelet degranulation and the immune system response within nasopharyngeal and urine samples derived from patients with COVID-19 (Akgun et al., 2020; Li et al., 2020). These results confirm the clinical data evidencing an innate immune response and subsequent cytokine storm during SARS-CoV-2 infection (Tay et al., 2020). Moreover, Messner et al. (2020) have demonstrated the utility of MS-based methods in clinical practice, reporting 27 potential serum biomarkers for COVID-19. Interestingly, these authors have applied short gradients combined with data-independent acquisition (SWATH-MS), achieving precise, reproducible, robust, and low-cost results, allowing 180 samples per day on a single mass spectrometer. Based on these studies, it is evident that the sensitivity and high throughput of specific MS approaches may complement the current clinical genetic studies based on the polymerase chain reaction, being a technological pillar to be considered for future assistance in tackling the worldwide spread of the novel coronavirus causing COVID-19.

## Effects of SARS-CoV-2 in Chemosensory Processing: What Role Can Proteomics Play?

**Figure 1:**
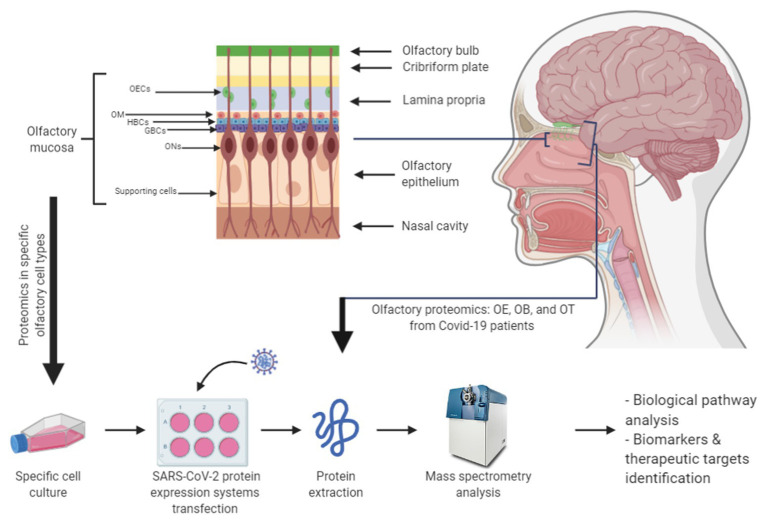
Potential olfactory proteomics workflows to characterize molecular disturbances caused by severe acute respiratory syndrome coronavirus 2 (SARS-CoV-2). The combination of commercial or primary cell lines from the olfactory mucosa with the transfection of expression systems for SARS-CoV-2 proteins (or infection with viral particles) represents a valuable approach to study the impact of SARS-CoV-2 infection in this region. In addition, the proteomic analysis of the OE-OB-OT axis from patients with COVID-19 will provide novel insights into the olfactory dysfunction triggered by SARS-CoV-2. GBC, globular basal stem cell; HBC, horizontal basal stem cell; OB, olfactory bulb; OE, olfactory epithelium; OEC, olfactory ensheathing cell; OM, olfactory mucosa mesenchymal stem cell; ON, olfactory neuron; OT, olfactory tract.

Emerging evidence shows that SARS-CoV-2 infection causes pleiotropic effects. Apart from the respiratory system, other tissues such as the small intestine, renal tubules, and arterial smooth muscle cells are vulnerable to SARS-CoV-2 infection because of their high expression of ACE2 (Zou et al., 2020). Among other symptoms, olfactory loss is the early predominant neurological symptom (Baig et al., 2020; Lechien et al., 2020; Moein et al., 2020; Parma et al., 2020). The nasal cavity is one of the routes of SARS-CoV-2 entry, where the presence of ACE2 and TMPRSS2 in stem cells and olfactory epithelial support cells is higher than that in olfactory sensory neurons (Brann et al., 2020; Ueha et al., 2020), suggesting that the first group may be partially responsible for the olfactory dysfunction observed in patients with COVID-19. Both ACE2 and TMPRSS2 genes are co-expressed with genes involved in innate immunity in nasal structures, indicating a potential role in battling the infection in the initial phases of the disease (Sungnak et al., 2020). Nevertheless, despite all this evidence, the molecular consequences of the entrance of the virus in the olfactory system remain unknown. Owing to the relevance of olfactory loss as an early predictor of COVID-19, the application of olfactory proteomics (Lachén-Montes et al., 2016) would shed new light on the potential mechanisms disrupted by SARS-CoV-2 at the level of: (i) the olfactory neuroepithelium (involved in odor perception), (ii) the olfactory bulbs and tracts (involved in the transmission of olfactory information), and (iii) the olfactory cortex (involved in odor recognition/discrimination and memorization). To do this, the scientific community requires autopsies of the brains of patients with COVID-19, detailed neuropathological examination, and initiatives aimed at biobanking of the olfactory epithelium and olfactory structures. However, for biosecurity reasons, the availability of this biological material is not well established. To partially solve this problem, the deployment of highly controlled *in vitro* systems that model different stations of the olfactory pathway in combination with large-scale MS-based quantitative proteomics will allow the molecular imbalance induced by SARS-CoV-2 in cellular and neuronal circuits in the central olfactory areas to be monitored (Figure 1).

## Future Directions

The urgent need for the scientific community to face the current SARS-CoV-2 world pandemic has evidenced the great efforts of proteomic scientists to decipher molecular knowledge about SARS-CoV-2 and COVID-19. In this mini-review, we have proposed analysis of the intranasal route of SARS-CoV-2 between the nose and the brain to improve our understanding of the early olfactory loss observed in patients with COVID-19. The application of single-cell proteomics or laser capture microdissection coupled with LC-MS/MS analysis will shed new light on the vulnerability of specific olfactory cell types through the specific characterization of deregulated proteomic maps triggered by SARS-CoV-2 infection. Alternative approaches simulating SARS-CoV-2 infection in olfactory cell lines could also be useful to study the progression of the viral infection using a wide range of proteomic strategies, identifying new viral-host protein interactions and potential therapeutic targets for non-invasive intranasal treatments. However, urgent and conjoined efforts from researchers, clinicians, and biosafety and ethics authorities are needed to facilitate the provision of clinical and autopsy biospecimens in order to clarify and understand the potential alterations that occur in the olfactory axis during COVID-19 progression.

## Author Contributions

ES: conceptualization. ML-M, FC, JF-I, and ES: formal analysis. FC, JF-I, and ES: funding acquisition. ES: supervision. ML-M and ES: writing – original draft. All authors contributed to the article and approved the submitted version.

### Conflict of Interest

The authors declare that the research was conducted in the absence of any commercial or financial relationships that could be construed as a potential conflict of interest.
